# Uncovering genetic and metabolite markers associated with resistance against anthracnose fruit rot in northern highbush blueberry

**DOI:** 10.1093/hr/uhad169

**Published:** 2023-08-20

**Authors:** MacKenzie Jacobs, Samantha Thompson, Adrian E Platts, Melanie J A Body, Alexys Kelsey, Amanda Saad, Patrick Abeli, Scott J Teresi, Anthony Schilmiller, Randolph Beaudry, Mitchell J Feldmann, Steven J Knapp, Guo-qing Song, Timothy Miles, Patrick P Edger

**Affiliations:** Department of Biochemistry and Molecular Biology, Michigan State University, East Lansing, MI 48824, USA; Department of Horticulture, Michigan State University, East Lansing, MI 48824, USA; Molecular Plant Science Program, Michigan State University, East Lansing, MI 48824, USA; Molecular Plant Science Program, Michigan State University, East Lansing, MI 48824, USA; Department of Plant, Soil and Microbial Sciences, Michigan State University, East Lansing, MI 48824, USA; Department of Horticulture, Michigan State University, East Lansing, MI 48824, USA; Department of Horticulture, Michigan State University, East Lansing, MI 48824, USA; Department of Horticulture, Michigan State University, East Lansing, MI 48824, USA; Department of Horticulture, Michigan State University, East Lansing, MI 48824, USA; Department of Horticulture, Michigan State University, East Lansing, MI 48824, USA; Department of Horticulture and Natural Resources, Kansas State University, Olathe, KS 66061, USA; Department of Horticulture, Michigan State University, East Lansing, MI 48824, USA; Genetics and Genome Sciences Program, Michigan State University, East Lansing, MI 48824, USA; Mass Spectrometry & Metabolomics Core, Michigan State University, East Lansing, MI 48824, USA; Department of Horticulture, Michigan State University, East Lansing, MI 48824, USA; Department of Plant Sciences, University of California, Davis, CA 95616, USA; Department of Plant Sciences, University of California, Davis, CA 95616, USA; Department of Horticulture, Michigan State University, East Lansing, MI 48824, USA; Molecular Plant Science Program, Michigan State University, East Lansing, MI 48824, USA; Department of Plant, Soil and Microbial Sciences, Michigan State University, East Lansing, MI 48824, USA; Genetics and Genome Sciences Program, Michigan State University, East Lansing, MI 48824, USA; Department of Horticulture, Michigan State University, East Lansing, MI 48824, USA; Molecular Plant Science Program, Michigan State University, East Lansing, MI 48824, USA; Genetics and Genome Sciences Program, Michigan State University, East Lansing, MI 48824, USA

## Abstract

Anthracnose fruit rot (AFR), caused by the fungal pathogen *Colletotrichum fioriniae,* is among the most destructive and widespread fruit disease of blueberry, impacting both yield and overall fruit quality. Blueberry cultivars have highly variable resistance against AFR. To date, this pathogen is largely controlled by applying various fungicides; thus, a more cost-effective and environmentally conscious solution for AFR is needed. Here we report three quantitative trait loci associated with AFR resistance in northern highbush blueberry (*Vaccinium corymbosum*). Candidate genes within these genomic regions are associated with the biosynthesis of flavonoids (e.g. anthocyanins) and resistance against pathogens. Furthermore, we examined gene expression changes in fruits following inoculation with *Colletotrichum* in a resistant cultivar, which revealed an enrichment of significantly differentially expressed genes associated with certain specialized metabolic pathways (e.g. flavonol biosynthesis) and pathogen resistance. Using non-targeted metabolite profiling, we identified a flavonol glycoside with properties consistent with a quercetin rhamnoside as a compound exhibiting significant abundance differences among the most resistant and susceptible individuals from the genetic mapping population. Further analysis revealed that this compound exhibits significant abundance differences among the most resistant and susceptible individuals when analyzed as two groups. However, individuals within each group displayed considerable overlapping variation in this compound, suggesting that its abundance may only be partially associated with resistance against *C. fioriniae*. These findings should serve as a powerful resource that will enable breeding programs to more easily develop new cultivars with superior resistance to AFR and as the basis of future research studies.

## Introduction

**Figure 1:**
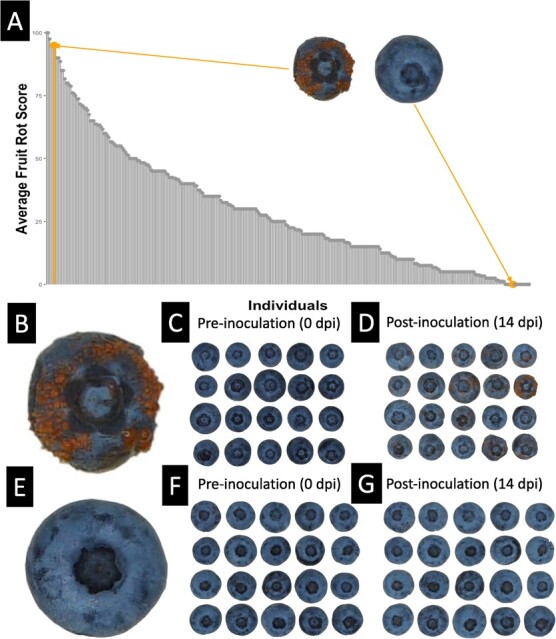
Susceptibility assessment of ‘Draper’ × ‘Liberty’ Hybrids to AFR. A. Susceptibility of hybrids to AFR. Bars highlighted in orange represent the average fruit rot susceptibility score of the individuals shown in panels (B) and (E). The average fruit rot score represents the average number of berries that developed fruit rot infection per each replicate of 5 berries. So, an average score of 100% demonstrates all berries developing infection. B. Infected fruit from a susceptible individual. C. Susceptible fruits prior to infection via *C. fioriniae*. D. Susceptible fruits 14 days after infection via *C. fioriniae*. E. Infected fruit from a susceptible individual. F. Resistant fruits prior to infection via *C. fioriniae*. G. Resistant fruits 14 days after infection via *C. fioriniae*.

Anthracnose fruit rot (AFR), caused by the fungal pathogen *Colletotrichum fioriniae* Marcelino & Gouli (=syn. *Glomerella acutata* var. *fioriniae*, formerly *Colletotrichum acutatum* Simmonds), is among the most destructive and widespread fruit disease of blueberries (*Vaccinium* sp.) [1]. The infection of *C. fioriniae* impacts fruit quality and can result in a complete loss of post-harvest yield [2]. *Colletotrichum* species have been reported to infect numerous other high-valued fruit crops, including apple, citrus, and strawberry [3]. Infections occur as early as fruit set but remain latent until the fruit ripens, complicating the disease’s detection and protection. Initially, sunken areas develop on the fruit surface, followed by the exudation of salmon-colored spore masses (Fig. 1).

**Figure 2:**
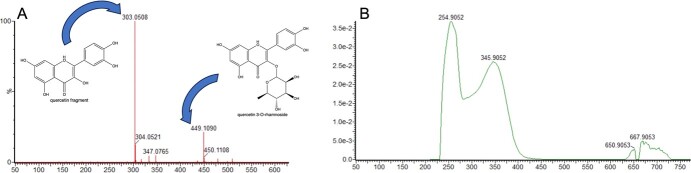
MS and UV/Vis Spectra of Quercetin Rhamnoside. (A) Ions detected consistent with expected m/z scores of a quercetin rhamnoside and its deglycosylated daughter ion. Here, the intact parent ion (right) is shown as quercetin 3-O-rhamnoside, however, the glycosylation may be in a different position. (B) UV/Vis spectra of the detected compound, consistent with the anticipated absorbance spectra of quercetin 3-O-rhamnoside.

Most blueberry cultivars are highly to moderately susceptible to AFR [4]. Fungicides remain the primary method to mitigate AFR infection in cultivated blueberry [5]. However, they are often expensive and not a favorable option for growers. Moreover, some of these fungicides are suspected carcinogens, whereas others are prone to fungicide resistance development [6]. Often, fungicide sprays are more frequently used than necessary because of the difficulty in optimizing spray timing due to the long latency period and variable weather conditions influencing the pathogen life cycle [5]. Therefore, the development of AFR-resistant cultivars is highly desired by the blueberry industry [7].

Several highly resistant cultivars have been identified, including northern highbush *Vaccinium corymbosum* L. ‘Draper’, which display strong resistance in the field and in laboratory inoculation studies [8, 9]. The genome of ‘Draper’ [10] was previously sequenced for three primary reasons: (1) it is a commonly utilized parent in breeding programs, (2) it is widely cultivated worldwide as an early mid-season ripening variety, and (3) it is highly resistant to AFR. However, to our knowledge, no cultivars exhibit complete resistance [11, 12]. In these studies [11, 12], *C. fioriniae* had differential infection strategies and infection rates in resistant versus susceptible cultivars. Furthermore, Miles and Hancock [4] recently reported that resistance to AFR infection is highly heritable and argue that there are likely only a few loci involved in resistance. However, the underlying genetic mechanism(s) of resistance to AFR remains poorly understood in blueberry and other fruit crops.

Blueberry fruits contain a high concentration of many phytochemicals, including compounds with known antifungal properties [13, 14]. One potential component of resistance to AFR could involve specialized metabolites. For example, quercetin 3-O-rhamnoside is a flavonol glycoside synthesized from the amino acid precursor L-phenylalanine via the phenylpropanoid pathway [15] whose antimicrobial activity has been demonstrated against *C. fioriniae* [13], *Pseudomonas maltophilia,* and *Enterobacter cloacae* [16]. In fact, treating susceptible blueberry fruits with a 4% solution of extract from resistant fruit containing quercetin 3-O-rhamnoside, among other anthocyanins and non-anthocyanin flavonoids, decreased *C. fioriniae* infection by 88% [13]. Quercetin and its glycosides have been studied in other systems, but the dynamics of these compounds remain poorly understood in blueberry.

Quercetin glycosides may be deglycosylated, leaving the bioactive core, quercetin [17]. Structural analysis of plant-derived flavonoids revealed that quercetin contains numerous structural components important in bioactivity against certain pathogens, including methicillin-resistant *Staphylococcus aureus*, vancomycin-resistant enterococci, and *Burkholderia cepacia* [18]. Furthermore, quercetin may be oxidized to form quinones, antifungal compounds previously shown to be effective against certain *Colletotrichum* species [19]. However, previous studies have also proposed that AFR resistance in ripe blueberries may be due to an interaction between simple phenolic compounds and organic acids and not necessarily individual fungitoxic compounds [20–22].

Here, we used a genetic mapping approach to identify genomic loci associated with resistance to AFR infection in northern highbush blueberry. We generated an RNAseq dataset to identify which genes are differentially expressed during infection in ‘Draper’ mature fruits. Finally, we performed metabolite profiling in mature fruits and identified a metabolite with properties consistent with a quercetin rhamnoside whose abundance is positively correlated with AFR resistance.

## Results

### Identification of genetic markers associated with AFR resistance

**Figure 3:**
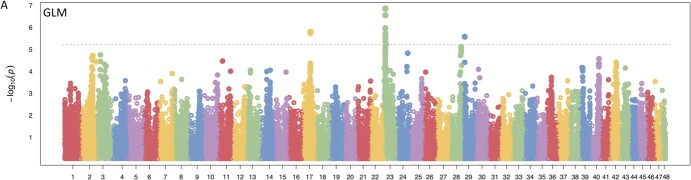
Genome-wide association study of AFR resistance in blueberry. Manhattan plot of SNP association with fruit rot resistance using a GLM model (Benjamini-Hochberg corrected significance threshold: 5.9e-6, dotted line). Four loci on chromosomes 17, 23 (2 loci; Supplemental Figure S6), and 29 show a significance beyond the FDR threshold.

Individuals within the ‘Draper’ × ‘Liberty’ genetic mapping population exhibited variation in susceptibility to AFR infection (Fig. 1). High resistance without showing any obvious AFR symptoms was observed in ~5% of the individuals surveyed. In comparison, ~4.7% exhibited high susceptibility to AFR—individuals averaging 4 or more out of 5 berries per replicate developing signs of rot (Fig. 1; Supplemental Figure S1; Supplemental Data S1, S2, andS3). Association mapping used genotyping data and fruit rot susceptibility scores as inputs into the GAPIT tool to determine the association of each SNP with the resistance phenotype under three models (GLM, BLINK, FarmCPU; Supplemental Figure S2). Overall, BLINK and FarmCPU models each suggested six QTLs after the Benjamini-Hochberg FDR adjustment, while the GLM model (Fig. 3; Supplemental Figure S3) suggested four distinct loci (12 distinct QTL loci over the three models). All approaches determined a significant QTL around a marker at Chromosome 17 position 22 625 275, while BLINK and GLM alone found a significant QTL at Chromosome 23 position 3 482 889, and an SNP on Chromosome 28 at position 31 421 447 was significant only in the BLINK and FarmCPU models (Fig. 3). The same regions were identified using R/qtl, with both a standard single QTL (qtl::scanone) and composite interval mapping (qtl::cim) and but with relatively lower LOD scores (Supplemental Figure S3). It’s important to note that Chromosomes 17, 23 and 28 are not homoeologous chromosomes, which are derived from the polyploidization event (Supplemental Figures S4 &S5).

The SNP on Chromosome 17 at position 22 625 275 lies in a candidate regulatory locus 1.8 Kilo bases (Kb) upstream of a poorly characterized protein with a moderate 34% similarity to the Arabidopsis cytokine signaling protein AT4G33800, a putative member of the SOB-FIVE- LIKE (SOFL) family of cytokinin genes [55]. SOFLs are a plant-specific gene family whose functions remain poorly understood even in model species such as Arabidopsis [55]. Cytokinins are plant hormones that have diverse functions, including various aspects influencing plant growth and development, but they are also known to impact immunity and resistance against pathogen infections [56].

On chromosome 23 at position 3 482 889, there is an SNP that lies somewhat upstream (6 Kb) of a gene model orthologous to the Arabidopsis YABBY family transcription factor (TF) gene AT2G26580 (Yab5) as determined by both gene synteny and protein similarity (BLASTp 75%). While the most significant marker in this locus is somewhat distal to the gene’s transcription start site (TSS), the association peak in the GLM model is broadly characteristic of a sweep and includes linked SNPs both upstream and downstream of the gene. The YABBY family of TFs, notably Yab5, have been reported to interact physically with elements in the jasmonate pathway and may be involved indirectly in pathogen defense [57, 58] and potentially acting as upstream regulators of phenylalanine ammonia-lyase (PAL) [59]. PAL is an early component of the quercetin 3-O-rhamnoside biosynthesis pathway, where it catalyzes the conversion of L-phenylalanine to cinnamate and ammonium [60], a step common to the biosynthesis of anthocyanins and proanthocyanidins as well [60].

The SNP located on Chromosome 28 at position 31 421 447 was flagged for its highly significant association with fruit rot resistance by both BLINK and FarmCPU approaches. This SNP is genic and one of three synonymous SNPs located at consecutive coding 4D sites in a gene model predicted by Augustus [61] starting at the highly conserved residue 186 and positioned in exons on either side of the splice junction for the terminal coding exon. The gene model is syntenic with and has 73% BLASTp identity to the Arabidopsis gene GGL17 (AT3G11210), an SGNH hydrolase-type esterase superfamily protein that is predicted to function in anthocyanin metabolic processes [62, 63].

### Assessment of differential gene expression in response to anthracnose infection

Gene expression changes were evaluated in the fruit of ‘Draper’ following inoculation with *C. fioriniae*. Among the 128 559 protein-coding genes annotated in the haplotype-phased tetraploid genome of ‘Draper’ [10], a total of 2948 DEGs were identified during any one of the five time points during infection between the treated fruit and the corresponding controls (SRA SUB12410201). The most DEGs were identified after 2 DPI (48 hrs) and the lowest after 1 hr following inoculation. The number of identified DEGs per stage are as follows: Day 0 (450 upregulated, 57 downregulated), Day 1 (793 upregulated, 189 downregulated), Day 2 (1423 upregulated, 284 downregulated), Day 3 (600 upregulated, 13 downregulated), and Day 4 (1016 upregulated, 95 downregulated). Most DEGs, 2559 out of 2948 (~87%), exhibit single rhythmic patterns.

First, we evaluated the functional enrichment for DEGs across all time points (134 genes total). Five KEGG pathways were identified as being enriched with significant DEGs; 1. ath01100 Metabolic pathways (FDR *P*-value = 0.0020), 2. ath00520 Amino sugar and nucleotide sugar metabolism (FDR *P*-value = 0.0087), 3. ath00944 Flavone and flavonol biosynthesis (FDR *P*-value = 0.0087), 4. ath00900 Terpenoid backbone biosynthesis (FDR *P*-value = 0.0471), and 5. ath01110 Biosynthesis of secondary metabolites (FDR *P*-value = 0.0471) (Table 1). No Gene Ontology (GO) Biological Processes terms were significantly enriched among these genes.

**Table 1:** KEGG pathway enrichment.

KEGG ID	Pathway description	Timepoint 1	Timepoint 2	Timepoint 3	Timepoint 4	Timepoint 5
ath01100	Metabolic pathways	1.50E-05	0.00018	5.27E-16	6.01E-09	3.21E-14
ath01110	Biosynthesis of secondary* metabolites	0.0015	4.98E-05	5.39E-17	6.01E-09	2.00E-14
ath00908	Zeatin biosynthesis	NS	0.028	0.008	0.0417	0.00095
ath00940	Phenylpropanoid biosynthesis	NS	0.0083	4.89E-05	NS	1.23E-05
ath00941	Flavonoid biosynthesis	NS	0.0019	0.0098	NS	0.0015
ath00945	Stilbenoid, diarylheptanoid and gingerol biosynthesis	NS	0.0048	0.0141	NS	0.0314
ath00480	Glutathione metabolism	NS	NS	0.0237	0.00026	0.00061
ath00900	Terpenoid backbone biosynthesis	NS	NS	4.89E-05	0.0152	0.0182
ath00500	Starch and sucrose metabolism	0.0477	NS	0.0461	NS	NS
ath00010	Glycolysis / Gluconeogenesis	NS	NS	NS	0.0152	0.0182
ath00592	alpha-Linolenic acid metabolism	NS	NS	0.0486	NS	0.0182
ath01200	Carbon metabolism	NS	NS	0.0276	NS	0.0332
ath01230	Biosynthesis of amino acids	NS	NS	0.0237	NS	0.0332
ath00270	Cysteine and methionine metabolism	NS	NS	0.0463	NS	NS
ath00460	Cyanoamino acid metabolism	NS	NS	0.0463	NS	NS
ath00330	Arginine and proline metabolism	NS	NS	0.0408	NS	NS
ath00520	Amino sugar and nucleotide sugar metabolism	NS	NS	NS	NS	0.0297
ath04016	MAPK signaling pathway	NS	NS	NS	NS	0.0314
ath00130	Ubiquinone and other terpenoid-quinone biosynthesis	NS	NS	NS	NS	0.0332
ath00904	Diterpenoid biosynthesis	NS	NS	NS	NS	0.0332

^*^Referred to as ‘specialized metabolites’ throughout.

**Figure 4:**
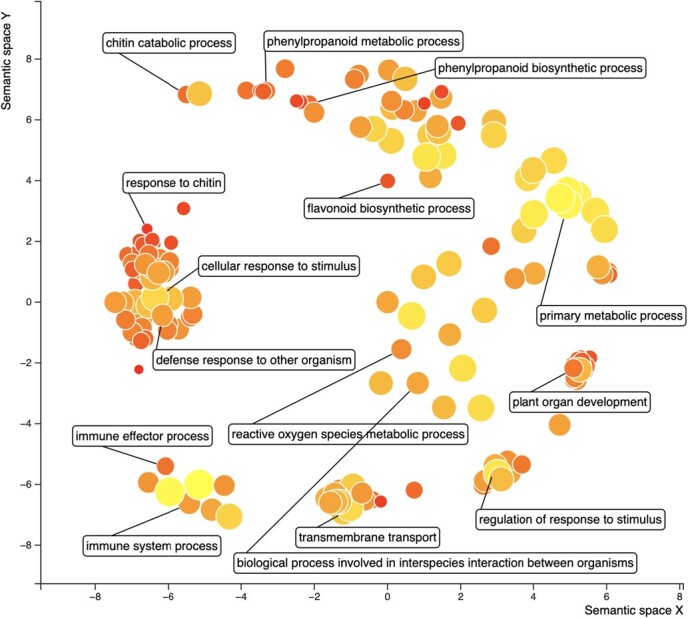
Scatterplot of significantly enriched gene ontology (GO) terms of differentially expressed genes in blueberry fruit following treatment with the fungal pathogen *C. fioriniae*. The scatterplot generated using Revigo [64] displays the relationship among GO terms identified among significantly differentially expressed genes following inoculation of blueberries with *C. fioriniae*. The size of the bubble representing each GO term indicates the frequency in the underlying GOA database, while hue indicates *P*-value significance (dark being most significant). Most highly similar GO terms form clusters. The identity of select GO terms are shown with arrows and boxes.

Next, we evaluated gene enrichment for differentially expressed genes across any time point. A total of 16 KEGG pathways were identified as significantly enriched with differentially expressed genes, including four of the five KEGG pathways identified when analyzing genes significantly differentially expressed during all time points (Table 1). The only KEGG pathway that was not enriched here was ‘ath00944 Flavone and flavonol biosynthesis’ but instead ‘ath00941 Flavonoid biosynthesis (FDR *P*-value = 0.0012)’, as well as the metabolite precursor biosynthetic pathways, ath00940 ‘Phenylpropanoid biosynthesis (FDR *P*-value = 0.0009)’ and ‘ath00360 Phenylalanine metabolism (FDR *P*-value = 0.0356), for quercetin 3-O-rhamnoside, was identified as being significantly enriched. A total of 183 GO terms for biological processes were identified as being enriched with significantly differentially expressed genes, including the majority being either related to stress (e.g. GO:0006950 Response to stress, FDR *P*-value = 9.59e-20), defense (e.g. GO:0006952 Defense response, FDR *P*-value = 2.41e-07) and/or metabolism (e.g. GO:0009698 Phenylpropanoid metabolic process, FDR *P*-value = 2.16e-08) (Fig. 4). The list of significantly differentially expressed genes identified here also significantly overlaps with genes identified by Toth et al. (2016) that investigated powdery mildew colonization in grape (FDR *P*-value = 9.40e-10) [65]. Similarly, there was a significant overlap of differentially expressed genes discovered here with those identified from previous studies that focused on reactive oxygen species (ROS) signaling (e.g. [66], FDR *P*-value = 3.84e-07), fungal pathogen resistance (e.g. [67], FDR *P*-value = 0.00031), biosynthesis of flavonol glycosides (e.g. [68], FDR *P*-value = 0.0012) and phenylpropanoid metabolism in response to fungal interactions (e.g. [69], FDR *P*-value = 0.0018) (Supplemental Data S6).

Lastly, we evaluated each of the individual time points. During the first time point (1 hr post-inoculation), three KEGG pathways and 56 GO biological process terms were identified as enriched with significantly differentially expressed genes. The three KEGG pathways are 1. Ath01100 Metabolic pathways (FDR *P*-value = 1.50e-05), 2. Ath01110 ‘Biosynthesis of secondary metabolites’ (FDR *P*-value = 0.0015) and 3. Ath00500 ‘Starch and sucrose metabolism’ (FDR *P*-value = 0.0477) (Table 1). Highly enriched GO terms include GO:0006950 ‘Response to stress’ (FDR *P*-value = 5.04e-05) and GO:0050896 ‘Response to stimulus’ (FDR *P*-value = 1.23e-05). In addition, these genes had an enrichment of PFAM protein domains associated with ‘innate immunity’ (FDR *P*-value = 0.0052) (Supplemental Data S6).

During the second time point (24 hrs post-inoculation), six KEGG pathways and 56 GO biological process terms were identified that were enriched significantly differentially expressed genes.The six KEGG pathways are 1. Ath01110 ‘Biosynthesis of secondary metabolites (FDR *P*-value = 4.98e-05), 2. Ath01100 ‘Metabolic pathways’ (FDR *P*-value = 0.00018), 3. Ath00940 ‘Phenylpropanoid biosynthesis’ (FDR *P*-value = 0.0083), 4. Ath00941 ‘Flavonoid biosynthesis’ (FDR *P*-value = 0.0019), 5. ath00945 ‘Stilbenoid, diarylheptanoid and gingerol biosynthesis’ (FDR *P*-value = 0.0048), and 6. Ath00908 ‘Zeatin biosynthesis (FDR *P*-value = 0.0280) (Table 1). Highly enriched GO terms include GO:0010200 ‘Response to chitin’ (FDR *P*-value = 1.18e-07), GO:0006952 ‘Defense response’ (FDR *P*-value = 3.22e-06), and GO:0009698 ‘Phenylpropanoid metabolic process’ (FDR *P*-value = 5.98e-05). In addition, these genes had an enrichment of PFAM protein domains associated with ‘stress response’ (FDR *P*-value = 6.07e-05), ‘chitin degradation’ (FDR *P*-value = 0.0337), and chitin-binding (FDR *P*-value = 0.0103). A significant overlap in shared differentially expressed genes was observed at this time point with Toth et al. (2016), which investigated powdery mildew colonization in grape [65] (FDR *P*-value = 2.5e-05) (Supplemental Data S6).

During the third timepoint (48 hrs post-inoculation), fifteen KEGG pathways and 158 GO biological process terms were identified that were enriched with significantly differentially expressed genes. The top three most significant KEGG pathways are 1. Ath01110 ‘biosynthesis of secondary metabolites (FDR *P*-value = 5.39e-17), 2. Ath01100 ‘metabolic pathways’ (FDR *P*-value = 5.27e-16) and 3. Ath00940 ‘Pheylpropanoid biosynthesis (FDR *P*-value = 4.89e-05) (Table 1). Flavonoid biosynthesis (Ath00941) is also enriched with significantly differentially expressed genes (FDR *P*-value = 0.0098). Highly enriched GO terms include 1. GO:0050896 ‘Response to stimulus’ (FDR *P*-value = 4.68e-19), 2. GO:0009698 ‘Phenylpropanoid metabolic process’ (FDR *P*-value = 4.66e-09), 3. GO:0051707 ‘Response to other organism’ (FDR *P*-value = 2.84e-07), 4. GO:0009699 ‘Phenylpropanoid biosynthetic process’ (FDR *P*-value = 4.18e-06), and 5. GO:0045087 ‘Innate immune response’ (FDR *P*-value = 7.11e-06). In addition, these genes had an enrichment of PFAM protein domains associated with ‘chitin degradation’ (FDR *P*-value = 0.009) and ‘plant defense’ (FDR *P*-value = 0.0267). Greater overlap in shared differentially expressed genes was observed in this timepoint with Toth et al. (2016) (FDR *P*-value = 7.86e-11) [65] (Supplemental Data S6).

During the fourth time point (72 hrs post-inoculation), six KEGG pathways and 61 GO biological process terms were identified that were enriched significantly differentially expressed genes. The six KEGG pathways are 1. Ath01110 ‘Biosynthesis of secondary metabolites’ (FDR *P*-value = 6.01e-09), 2. Ath01100 ‘Metabolic pathways (FDR *P*-value = 6.01e-09), 3. Ath00480 ‘Glutathione metabolism’ (FDR *P*-value = 0.00026), 4. Ath00900 ‘Terpenoid backbone biosynthesis’ (FDR *P*-value = 0.0152), 5. Ath00010 ‘Glycolysis / Gluconeogenesis’ (FDR *P*-value = 0.0152) and 6. Ath00908 ‘Zeatin biosynthesis’ (FDR *P*-value = 0.0417) (Table 1). Highly enriched GO terms include 1. GO:0050896 ‘Response to stimulus’ (FDR *P*-value = 4.63e-09), 2. GO:0051707 ‘Response to other organism’ (FDR *P*-value = 0.00016), 3. GO:0045087 ‘Innate immune response’ (FDR *P*-value = 0.00096), 4. GO:0009699 ‘Phenylpropanoid biosynthetic process’ (FDR *P*-value = 0.0024), and 5. GO:0006952 ‘Defense response’ (FDR *P*-value = 0.0197). In addition, these genes had an enrichment of PFAM protein domains associated with ‘chitin-binding’ (FDR *P*-value = 0.0048) and ‘chitin degradation’ (FDR *P*-value = 0.0153). A significant overlap, but lower than compared to the previous time-point, in shared differentially expressed genes was observed at this timepoint with Toth et al. (2016) (FDR *P*-value = 1.28e-08) [65] (Supplemental Data S6).

During the final time point (96 hrs post-inoculation), 16 KEGG pathways and 112 GO biological process terms were identified that were enriched significantly differentially expressed genes. The top three most significant KEGG pathways are 1. Ath01110 ‘biosynthesis of secondary metabolites (FDR *P*-value = 2e-14), 2. Ath01100 ‘metabolic pathways’ (FDR *P*-value = 3.21e-14) and 3. Ath00940 ‘Pheylpropanoid biosynthesis (FDR *P*-value = 1.23e-05) (Table 1). Flavonoid biosynthesis (Ath00941) is also enriched with significantly differentially expressed genes (FDR *P*-value = 0.0015). The overall patterns observed for this time point are similar to the third time point. Highly enriched GO terms include 1. GO:0050896 ‘Response to stimulus (FDR *P*-value = 5.15e-16), 2. GO:0009698 ‘Phenylpropanoid metabolic process’ (FDR *P*-value = 1.15e-09), 3.. GO:0009699 ‘Phenylpropanoid biosynthetic process’ (FDR *P*-value = 3.13e-07), 4. GO:0009813 ‘Flavonoids biosynthesis process’ (FDR *P*-value = 0.00043) and 5. GO:0045087 ‘Innate immune response’ (FDR *P*-value = 0.0014) (Supplemental Data S6).

### Variation in metabolite abundance between resistant and susceptible individuals

In the initial screening, one replicate of fruit extracts from six resistant (average fruit rot score = 0%) and six susceptible (average fruit rot score ≥ 80%) individuals from the genetic mapping population were analyzed in an untargeted manner using liquid chromatography coupled with mass spectrometry (LC–MS). We compared the chromatograms to identify peaks with the largest fold changes between the resistant and susceptible individuals. A peak annotated as the quercetin daughter fragment of quercetin rhamnoside showed the largest fold change at ~2.5-fold higher abundance in resistant lines. This finding is supported by work published by Miles et al. (2013), stating that quercetin 3-O-rhamnoside extracted from blueberry fruit exhibits antifungal properties and is effective against the causative agent of fruit rot [13].

**Figure 5:**
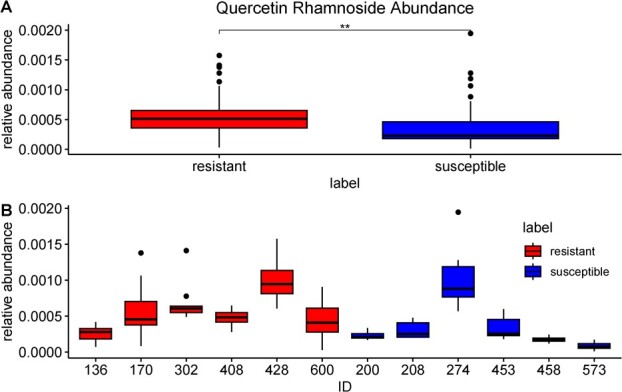
Abundance of metabolic markers in resistant and susceptible fruits. Normalized abundances of the suspected quercetin rhamnoside compound by fruit rot susceptibility (A) and by individual (B). Throughout the figure, boxes represent the interquartile ranges, bold horizontal lines depict the median of the observed abundances, vertical lines represent upper and lower extremes, and individual points represent outliers.

To further explore the potential role of the suspected quercetin rhamnoside in AFR resistance, targeted metabolite analysis was performed with additional replicates for each individual to quantify the abundance of the suspected quercetin rhamnoside in these extracts. Once again, we observed a significantly higher concentration of suspected quercetin rhamnoside in the extracts of resistant lines compared to susceptible lines (Fig. 5). However, some susceptible individuals did contain levels of these compounds more similar to resistant individuals (Supplemental Data S4, S5).

## Discussion

AFR is a top disease priority for the blueberry industry, as it can result in up to 100% post-harvest yield loss [2, 70]. Thus, growers have largely relied on fungicides to maximize yields. Both the infection and resistance mechanisms of AFR are highly variable among and within crops (see review [71]). Resistance may arise from passive mechanisms such as physiological fruit characteristics and pre-existing compounds with antifungal properties. Immature fruits often exhibit many features that lend themselves to resistance to anthracnose, such as firmness [72, 73], pH [74], and antimicrobial compounds [75]. However, these resistance factors tend to dampen as fruit matures [72, 73]. Further, the accumulation of soluble sugars in conjunction with ascorbic acid was previously associated with anthracnose resistance in guava [76]. Work in blueberry indicates a connection between sugar content and anthracnose resistance, but some moderately susceptible cultivars have high sugar concentrations [71]. This suggests that sugar content may be only one piece of a multi-factor resistance mechanism. Additionally, the abundance of certain fruit volatiles, including (E)-Hex-2-enal, has been linked to fruit rot resistance in strawberry [77]. While some of these volatiles are also found in blueberry, their presence and quantity are not correlated with resistance [8, 78]. Various antimicrobial compounds are correlated with AFR resistance, including 1-acetoxy-2-hydroxy-4-oxoheneicosa-12,15-diene [75] and avocadynone acetate [79] in avocado, and dopamine and dopamine-derivatives in banana [80]. In blueberry, resistance was previously hypothesized to come from the interaction of several compounds, including phenolics and organic acids [20]. Supportive of this hypothesis, two non-anthocyanin flavonoids from blueberry fruit, quercetin 3-O-rhamnoside and putative syringetin-rhamnoside, were found to have antifungal activity against the causative agent of AFR [13].

Alternatively, resistant fruits may prompt active resistance mechanisms such as inducible defense-related proteins, including chitinases and β-1-3-glucanases that degrade the cell wall [81–84]. Expression of defense-related proteins in response to anthracnose fungi has been previously reported in pepper [85–88], apple [89, 90], raspberry [91], tomato [92], and blueberry [11] . Putative defense genes in blueberry include those that encode cell wall degrading protein, pathogenesis-related protein 10 (PR10), metallothionein-like protein, and monodehydroascorbate reductase [11]. Moreover, resistant fruits may actively combat anthracnose infection through the formation of reactive oxygen species (ROS). Production of ROS in response to anthracnose has been documented in tomato [13, 93], strawberry [94], and blueberry [12, 13, 93]. In all three crops, ROS occurs at or near the time of attempted penetration of the pathogen. Concurrently, infected fruits upregulate expression of oxidative stress response genes to minimize potential harmful effects of the ROS on the host’s own tissues [11].

In this study, we sought to identify genomic loci associated with resistance to AFR by examining a genetic mapping population with tremendous variation in susceptibility. In order to capture allele and subgenome-specific variation that might be associated with resistance, we mapped to a fully haplotype resolved genome assembly with no explicit association made between the locations of the equivalent bases in the different subgenomes/haplotypes. Cultivated highbush blueberry (*V. corymbosum*) has a rich history of introgression from multiple other wild blueberry species from breeding efforts, plus natural gene flow that has previously shown to occur among sympatric blueberry species [95]. We have an unpublished analysis, as part of another study, that suggests that the genome of cultivar ‘Draper’, one of the parents analyzed here, has minimally 23% introgressed from at least three other *Vaccinium* species. This may, in part, be the reason why we identified significant candidate genomic regions in only one of the homoeologous chromosomes. Homoeologous chromosome sets are shown in Supplemental Figure S5. The different homoeologous chromosomes (i.e. haplotypes) of ‘Draper’ exhibit high sequence divergence. In effect, the tetraploid was treated in alignment terms as a single fully expanded haploid.

QTL mapping tools are available that function for polyploids, but they frequently depend on other tools such as polymap [96] that require dosage information that we are not exploring in the current work or require rather specific types of population that were not generated as a part of this study. Meanwhile, the GWAS approaches are necessarily somewhat robust to variable levels of population relatedness, explicitly incorporating this in approach-specific ways [26, 27], while more recent GWAS approaches adopt elements of QTL mapping to improve their sensitivity. Both the FARMCPU approach and BLINK approach for example exploit the generation of pseudo-markers and marker aggregation, FARMCPU using block-interval marker aggregation with block-size determined by MLE and BLINK marker aggregation in discovered bayesian linkage groups to recover essentially much of the power lost in approaches such as GWAS GLM/MLM where markers are considered essentially individually. Nonetheless, the different GWAS approaches selected very similar sites with only the association strength being notably different. BLINK and FARMCPU in this respect achieved higher levels of significance at these consistent sites likely due to their mapping-like marker aggregation. We have added to the supplementals a QTL mapping study results that includes standard interval mapping, composite interval mapping, and multiple QTL mapping to test whether the QTL and GWAS approaches are essentially in agreement. The QTL approaches largely support the GWAS sites albeit with the limitations on marker selection discussed above not always leading to greatly enhanced levels of significance relative to FARMCPU/BLINK (Supplemental Figures S2 andS3). In addition, we reran GWAS and QTL analysis to examine results with a binary categorization of susceptibility (<=1:0, >1:1). There was no evident increase in association power when classifying the genotypes in a binary form (Supplemental Figure S7).

We identified three loci located on pseudomolecules 17, 23, and 28 in the *V. corymbosum* ‘Draper’ genome that are significantly associated with the resistance phenotype (Fig. 3). Having several candidate causal SNPs is consistent with a polygenic resistance trait that may partly contribute to the observed continuous fruit-rot susceptibility distribution. Variants in several linked biosynthesis pathways, including cytokines, anthocyanins, and other flavonoids such as quercetin, may have become established in the population due to a protective role gained over time against multiple fungal pathogens. The identification of these loci will be useful for developing a molecular marker-assisted selection protocol for blueberry, which will greatly facilitate future screening for AFR resistance early in the breeding and selection process. Several candidate genes within these QTLs were previously associated with resistance against pathogens and/or flavonoid biosynthesis, including anthocyanins.

In comparing gene expression of blueberry fruits infected with *C. fioriniae* to uninfected fruits, numerous biological processes showed differential expression across the infection time course. Within 1 hour of infection, infected samples began upregulating genes related to metabolic pathways, including starch and sucrose metabolism and biosynthesis of secondary metabolites. In the following days, genes involved in metabolic pathways and biosynthesis of secondary metabolites continued to be upregulated. Additionally, the biosynthesis and metabolism of other primary and specialized metabolites and plant hormones were upregulated. Notably, many of the differential KEGG pathways show associations with the metabolite marker quercetin 3-O-rhamnoside. Most clearly, phenylpropanoid and flavonoid biosynthesis can be tied directly to quercetin 3-O-rhamnoside content as this metabolite falls within the phenylpropanoid and flavonoid families of specialized metabolites. Furthermore, the biosynthesis of amino acids may also be connected to quercetin 3-O-rhamnoside, as L-phenylalanine, an amino acid, is a precursor of quercetin 3-O-rhamnoside.

Our transcriptome analyses identified an enrichment of significantly differentially expressed genes associated with certain specialized metabolic pathways (e.g. flavonol biosynthesis) and pathogen resistance. Plus, several candidate genes within these QTLs were previously associated with resistance against pathogens and/or flavonoid biosynthesis, including anthocyanins. However, it’s important to note that none of these genes were identified as significantly differentially expressed. Low expressed genes, including certain regulators (e.g. transcription factors), may not be identified as significantly differently expressed but can cause a cascade of transcriptional changes, including inducing certain genetic pathways, and impact certain phenotypic traits. Thus, it’s possible that our transcriptome analyses were unable to identify them based on the current data and significance thresholds. We hope that the community will engage in combining these results with their own to further explore the data and identify additional potential candidate genes.

Lastly, we identified a flavonol glycoside with accurate mass, fragmentation, and absorbance characteristics consistent with a quercetin rhamnoside, whose abundance is significantly greater in berries from resistant individuals. These findings confirm data previously reported by Miles et al. (2013), which identified quercetin 3-O-rhamnoside as an antifungal component of blueberry fruit [13]. Several efforts were taken to normalize any variation in metabolite content due to fruit location on the bush, collection time, and fruit ripeness. Fruits were collected from the entire bush and randomly separated into three subsets. Fruits within each subset were ground together and homogenized, resulting in three separate homogeneous mixtures of powdered fruit from each individual. Variability of the quercetin rhamnoside abundances was observed within resistant and susceptible individuals (Fig. 5). Furthermore, levels of quercetin were previously shown to vary greatly between different harvests - indicating that the biosynthesis of these compounds may be influenced by the environment [97].

In general, flavonol glycosides are known to have antioxidant activity (even higher than anthocyanin antioxidant properties [98]), and blueberry fruit flavonol extracts demonstrate antioxidative activity against peroxyl and superoxide anion radicals [99]. Taken with the hypotheses of Cipollini and Stiles (1992a, 1992b, 1993), these findings suggest that quercetin 3-O-rhamnoside, a phenolic compound, is likely a major component of resistance to AFR in blueberry, but not the only component. Perhaps this molecule acts with other resistance mechanisms to protect the fruits from AFR.

Traditional breeding efforts in blueberry have contributed to major improvements of various target traits, but it is a lengthy and expensive process for perennial crops [31]. The use of molecular markers to guide breeding efforts has long been shown to greatly accelerate the development of superior cultivars, including selecting disease-resistant individuals [100]. There has been a strong community effort to develop and implement cost-effective methods for blueberry breeding programs [70]. Collectively, we hope that these findings reported in this study will allow breeders to develop new resistant cultivars to AFR more efficiently and rapidly by leveraging these new genetic regions to identify and select resistant individuals in their breeding programs. The phenotype predictive power from the combination of the three markers on chromosomes 17, 23 and 28 was assessed using a multiple regression approach. Individually the Pearson product moment (r) between phenotype on the 0–5 scale and genotype ranged from a low of 0.25 (chromosome 17 SNP) to a high of 0.30 (chromosome 23 SNP), jointly a predictive model generated from all 3 sites had an r of 0.49 (p ~ 0, dF = 3, F = 30.38). In summary, to the best of our knowledge, this is the first study to identify potential markers associated with resistance to AFR in blueberry. We anticipate that additional markers and candidate genes will likely be identified as part of future studies of this important target trait in blueberry. We see the research presented in this manuscript as a stepping stone toward uncovering the underlying mechanism(s) that contributes to anthracnose resistance in fruit of northern highbush blueberry.

## Materials and methods

### Plant materials

A genetic mapping population (F1) of northern highbush blueberry cultivars was derived from a cross between cultivars ‘Draper’ and ‘Liberty’. A total of 323 individual bushes growing at the Michigan State University Horticulture Teaching and Research Center were used during the 2020 and 2021 growing seasons in this study.

### Phenotyping - infection assay

To determine susceptibility to AFR, 5 to 20 full ripe blueberry fruits from the 323 F1 bushes were collected and assayed during July and August of 2020 (Supplemental Data S1) and 2021 (Supplemental Data S2). Fruits were sprayed evenly with *C. fioriniae* at a spore suspension of 1 x10^6^/mL, placed in humidor trays under ambient conditions (21°C 12 h:12 h light: dark), and inoculated fruits were monitored for signs of anthracnose infection 12 days post-inoculation (DPI). Fruits were assigned, prior to the inoculation, to replicates containing 5 berries each, and the number of berries that developed symptoms of infection per replicate was assessed (Fig. 1). A replicate score of 100% indicates that all berries developed visible infection symptoms. All replicate ‘scores’ from 320 individuals were averaged to produce the ‘average fruit rot scores’ shown in Fig. 1**(**Supplemental Data S3**)**.

### Genotyping

Genotyping for single nucleotide polymorphisms (SNPs) was performed by RAPiD Genomics (Gainesville, FL, USA) using the Capture-Seq targeted system. A total of 31 063 biotinylated probes of 120-mer were originally designed based on the ‘W8520’ draft genome v. 2013 [23]. The probe sequences were then remapped against the ‘Draper’ genome [10] to retrieve probes that strictly align to only a single homoeologous chromosome group [24]. From the filtered set, 10 000 probes were selected throughout the 12 largest haploid chromosomes set and small contigs to be used for targeted sequencing. The presence of SNPs in the selected probes was tested on the parental ‘Draper’ and ‘Liberty’ before sequencing the entire population. Sequencing was then carried out on the Illumina HiSeq platform using a 150 cycle paired-end layout, generating just over 200GBase of trimmed read data around the 10 000 probe sites. After read-trimming26 and variant calling27 SNPs were filtered with VCFTools [25] with parameters —min-meanDP 5 —minQ 30 —maf 0.05 —remove-indels —max-missing 0.25 —min-alleles 2 —max-alleles 2 —recode. Downstream analyses only analyzed samples with at least 10× sequence depth and this comprised 323 samples with phenotype data and a mean sequencing depth of 79.14× (395.7 Mb of data).

### QTL identification

A genome-wide association study was performed using the genotyping data and fruit rot susceptibility scores, with potential quantitative trait loci (QTL) associations assessed using the GAPIT tool (version 3) [26]. Several models were used to account for kinship and population structure (see the GAPIT manual for full model details: https://zzlab.net/GAPIT/gapit_help_document.pdf). Overall Q-Q plots suggested that GLM weakly compensated for demographic artifacts, while the FarmCPU [27]and BLINK [28] models both appeared to work well. Concordance between the most significant markers after false discovery rate (FDR: Benjamini and Hochberg [29]; < 0.05) correction in the association models was used to prioritize QTL locations for further investigation [30].

Genome wide association study was chosen over QTL mapping for several reasons. Northern highbush cultivars, including Draper and Liberty, are highly heterozygous and large amounts of introgression from multiple other wild blueberry species [31]. Thus, as part of this study, our goal was to analyze each haplotype separately, as a single fully expanded haploid. QTL mapping tools, which analyze allele dosage, are available for polyploid organisms, but this is outside of the scope of this project. Further, recent GWAS analysis methods utilize elements of QTL mapping to improve sensitivity. Here, we performed GWAS analysis using GLM, MLM, BLINK, and FarmCPU models as well as a QTL mapping study (R/qtl) that includes standard interval mapping, composite interval mapping, and multiple QTL mapping to test whether the QTL and GWAS approaches are in agreement (Supplementary Methods QTL mapping).

### RNA sequencing

Ripe berries were collected from ‘Draper’ and inoculated with either *C. fioriniae*, 1×10^6^ spores per milliliter with sterile distilled water, or sterile distilled water to serve as the ‘mock-inoculated’ control. Five time points were collected: one hour after inoculation (0 days) and 1, 2, 3, and 4 DPI for both inoculated and mock-inoculated with three biological replicates each (30 libraries total). Fruit tissue was sampled using a single-edged razor blade to excise all tissue within 2–3 mm of the calyx. For each of the 30 samples, RNA was extracted using the MagMax™ plant RNA Isolation Kit (Applied Biosystems/ThermoFisher Scientific) from ~50 mg of frozen ground tissue according to the manufacturer’s recommended protocol for use with the KingFisher™ Flex Magnetic Particle Processor 96DW (ThermoFisher Scientific). RNase-free PVP40 (2% w/v) was added to the lysis buffer according to the manufacturer’s recommendation to deal with polyphenol- and polysaccharide-rich samples. On-Column DNase digestion with the RNase-free DNase Set was used to remove DNA in the RNA samples (Qiagen, Valencia, CA, USA). RNAseq libraries were prepared using the mRNA HyperPrep Kit for Illumina® Platforms and the Dual-Indexed Adapter Kit for Illumina platforms and then sequenced using 150-bp paired-end reads on an Illumina HiSeq4000 system in MSU Research Technology Support Facility Genomics Core.

A total of 188.77 billion bases was sequenced, with an average of 20.97 million 150 bp paired end read per library (30 libraries total). Read quality was assessed using FastQC [32]. All reads were processed to remove adapters, low-quality leading or trailing bases, and minimum length using Trimmomatic v0.38 [33] with the following options: ILLUMINACLIP:TruSeq3-PE-2.fa:2:30:10:8:TRUE LEADING:3 TRAILING:3 SLIDINGWINDOW:4:15 MINLEN:36. Paired reads were aligned using Bowtie2 [34] against the ‘Draper’ reference genome and abundances estimated by the Expectation Maximum (RSEM) method [35]. The R package edgeR [36–46] (R version 3.4.1, edgeR version 3.18.1) was used to identify significantly differentially expressed genes (DEGs) following FDR correction (*P* < 0.05) to determine differential expression between the control and inoculated berries. Gene enrichment analyses were formed using ortholog predictions to *Arabidopsis thaliana* [47]*,* based on a combination of synteny- [48, 49] and BLASTp [50, 51] based approaches, and the STING database [52] containing KEGG pathway [53] and Gene Ontology [54] data for Arabidopsis [33] (Supplemental Data S6).

### Metabolite analysis

Extraction buffer was prepared by combining 800 mL of HPLC grade methanol (Sigma-Aldrich, ≥99.9%), 200 mL of HPLC grade H_2_O, 1 mL of formic acid, and 1 mL of 100 μM telmisartan (internal standard). Ripe blueberry fruits from the 12 individuals exhibiting the strongest resistance or susceptibility to AFR in the 2020 fruit rot scoring were collected in 2021 and frozen (Supplemental Table S1). Frozen berries were ground to powder with a small amount of dry ice using a mortar and pestle in liquid nitrogen to prevent melting. Portions of ground tissue were transferred to 2 mL snap tubes, and 1 mL of extraction buffer was added to each tube. Tubes were vortexed until the tissue was thawed and then centrifuged at 13 × g for 10 minutes to pellet insoluble material. Supernatants were decanted and diluted 1:1 in HPLC grade H_2_O + 0.1% formic acid. Snap tubes containing the pelleted insoluble material were dried at 60°C for one week (complete dryness), and the dry mass of extracted tissue was determined. Diluted extracts were analyzed on a Waters Acquity UPLC System coupled to a Waters Xevo G2-XS quadrupole time-of-flight mass spectrometer. Separations were performed using an Acquity UPLC HSS-T3 (1.8 μm; 2.1 × 100 mm) at 40°C. Solvent A consisted of H_2_O + 0.1% formic acid, and solvent B was acetonitrile. A 10-minute elution gradient was used to separate 10 μL of injected sample as follows (%A/%B): 0.00 min (100/0), 0.50 min (100/0), 6.00 min (50/50), 6.50 min (1/99), 8.50 min (1/99), 8.51(100/0), 10.00 (100/0) [10]. Positive ionization mode was used with a capillary voltage of 3.0 kV. Analysis of metabolites was completed in two phases. First, chromatograms and spectra were imported into Progenesis QI (Nonlinear Dynamics) which identifies peaks and quantifies them through the summation of intensities of compound adducts. The reported abundances for compounds in each sample were normalized to the dry mass of the extracted blueberry fruit tissue. The fold change of normalized metabolite intensities between resistant and susceptible lines was compared. The compound with the largest fold change, whose identity could be speculated, was consistent with the quercetin daughter fragment of a parent ion with qualities consistent with a quercetin rhamnoside (Fig. 2). This daughter ion has the same retention time as the quercetin rhamnoside parent, indicating that fragmentation likely occurred during ionization. The suspected quercetin rhamnoside was chosen for further pursuit. Next, the second round of LCMS analysis was performed on additional biological and technical replicates from each individual. A targeted QuanLynx processing method was designed to determine the intensity of the quercetin rhamnoside parent ion and the internal standard telmisartan (Supplemental Data S4). Parent ion intensities were normalized to telmisartan intensities and the dry mass of the tissue used to prepare the extract.

## Supplementary Material

Web_Material_uhad169
