# Supplementary material for: Uncovering genetic and metabolite markers associated with resistance against anthracnose fruit rot in northern highbush blueberry
Source: Hortic Res. 2023 Aug 20;10(10):uhad169. doi: 10.1093/hr/uhad169 (PMC10660357; doi:10.1093/hr/uhad169)
Supplement: Web_Material_uhad169 [file web_material_uhad169.zip › AFR_BB_2023_Supplement_V2.docx]

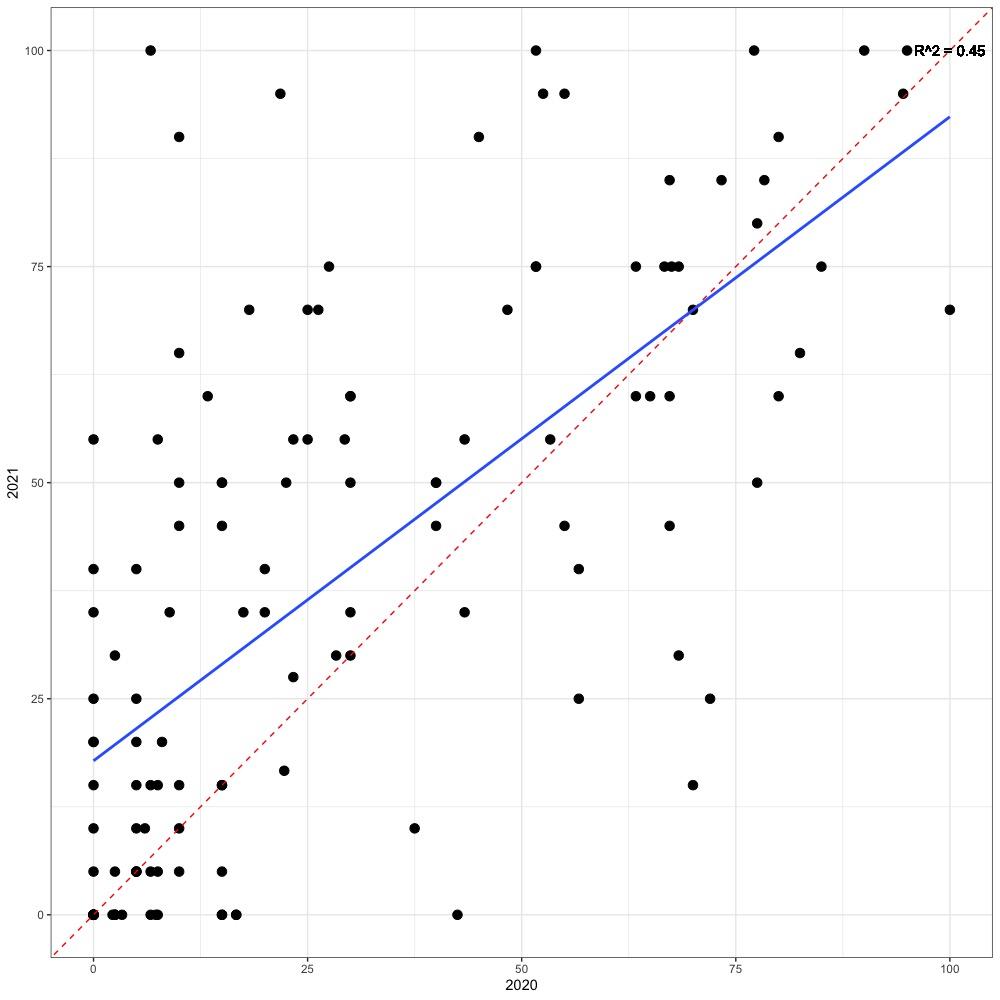


**Supplemental Figure 1.** Comparison of Anthracnose fruit rot susceptibility between 2020 and 2021 analyses. Each point represents an individual that was analyzed in both years. The red dashed line represents a theoretical 1:1 trendline, representing no variation between seasons. The blue solid line represents the actual trendline that fits the actual relationship between 2020 and 2021 susceptibilities.


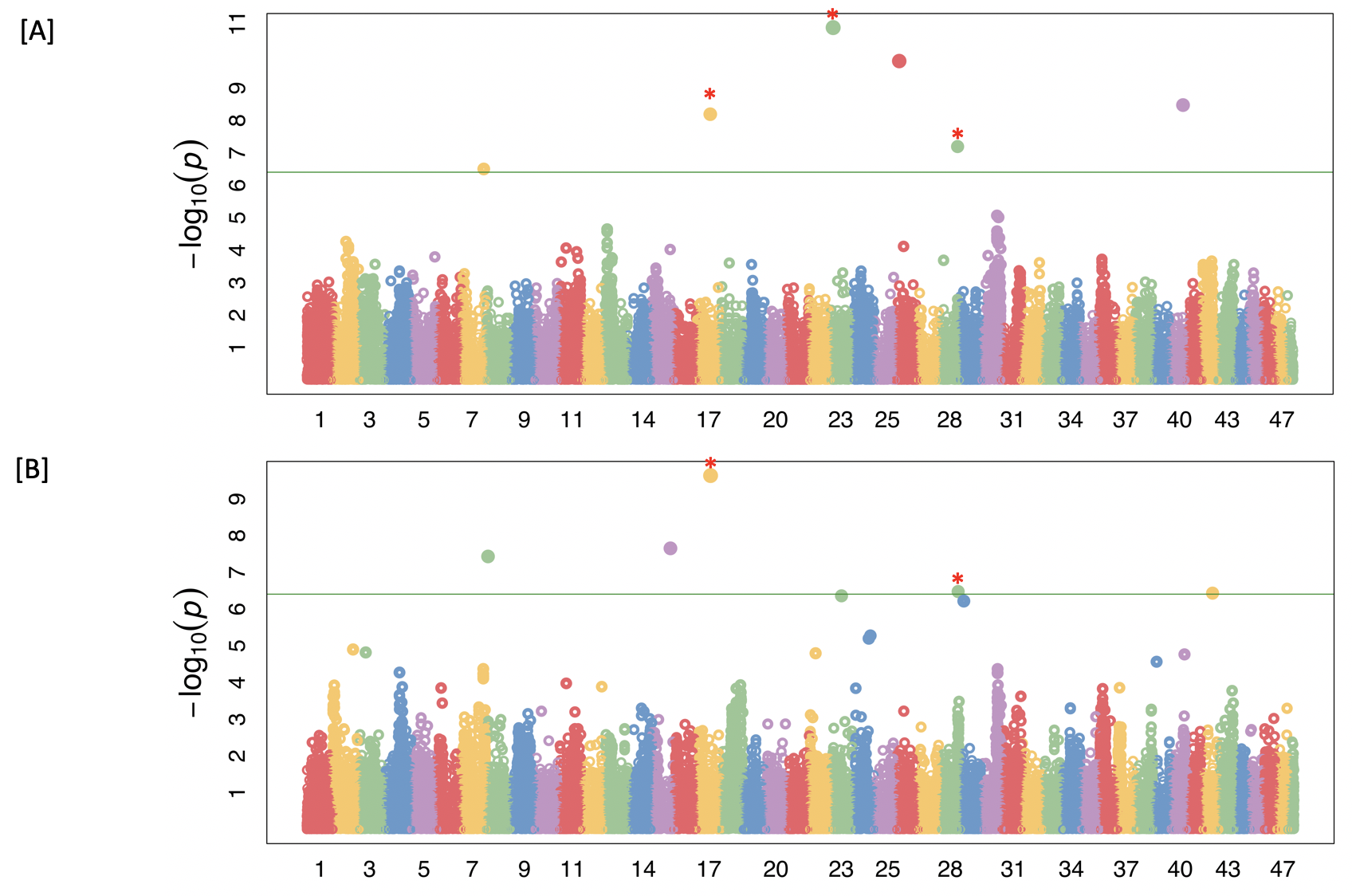


**Supplemental Figure 2:** Manhattan plots for (A) BLINK and (B) FARMCPU GAPIT models. Results from GLM are shown in FIgure 2. Red stars mark consensus sites (discovered by at least 2 of 3 GWAS models).


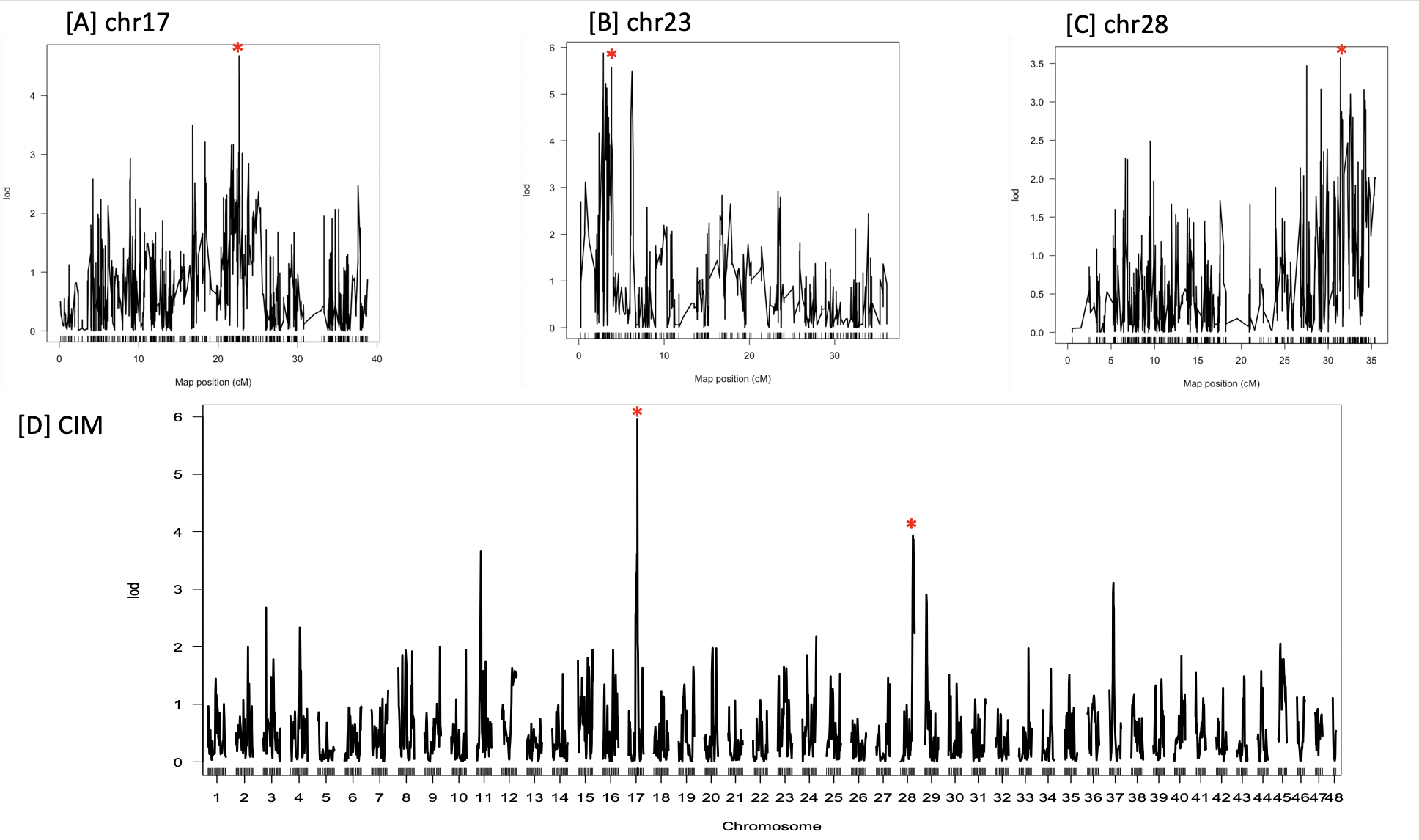


**Supplemental Figure 3:** Correspondence between the three consensus GWAS locations on chromosomes 17, 23 and 28 with individual QTL peaks (A-C) when using qtl::scanone to generate a standard single QTL model, and (D) using qtl::cim to develop a composite interval mapping model. Red stars indicate the position of consensus GWAS markers (those found in at least 2 of the 3 GWAS approaches) overlaid on the QTL lod-plots.


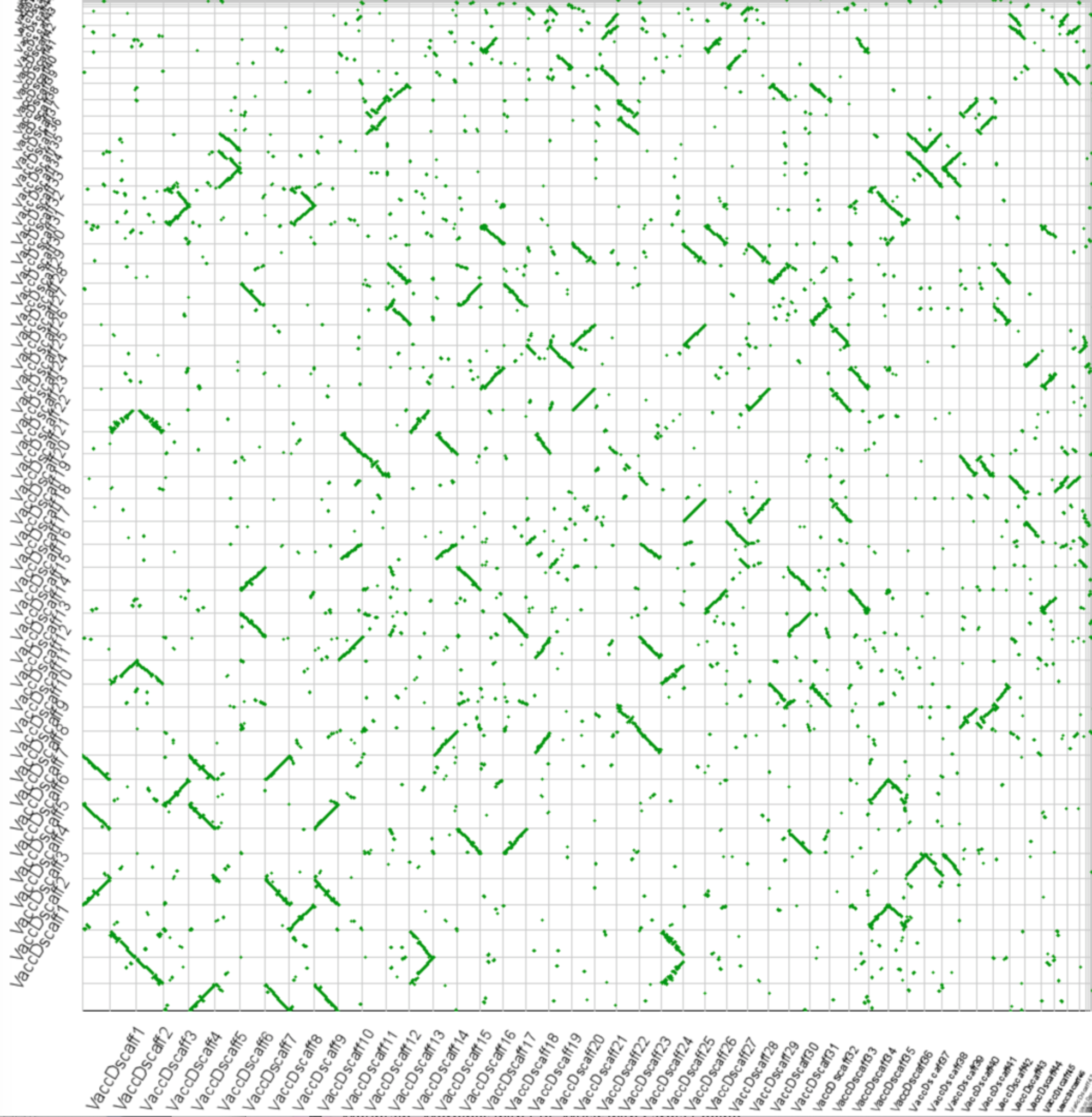


**Supplemental Figure 4:** Self-Self Syntenic map of the reference genome ‘Draper’ using CoGe – a platform for comparative genomics. This analysis can be regenerated using this weblink: <https://genomevolution.org/r/12w9o>.


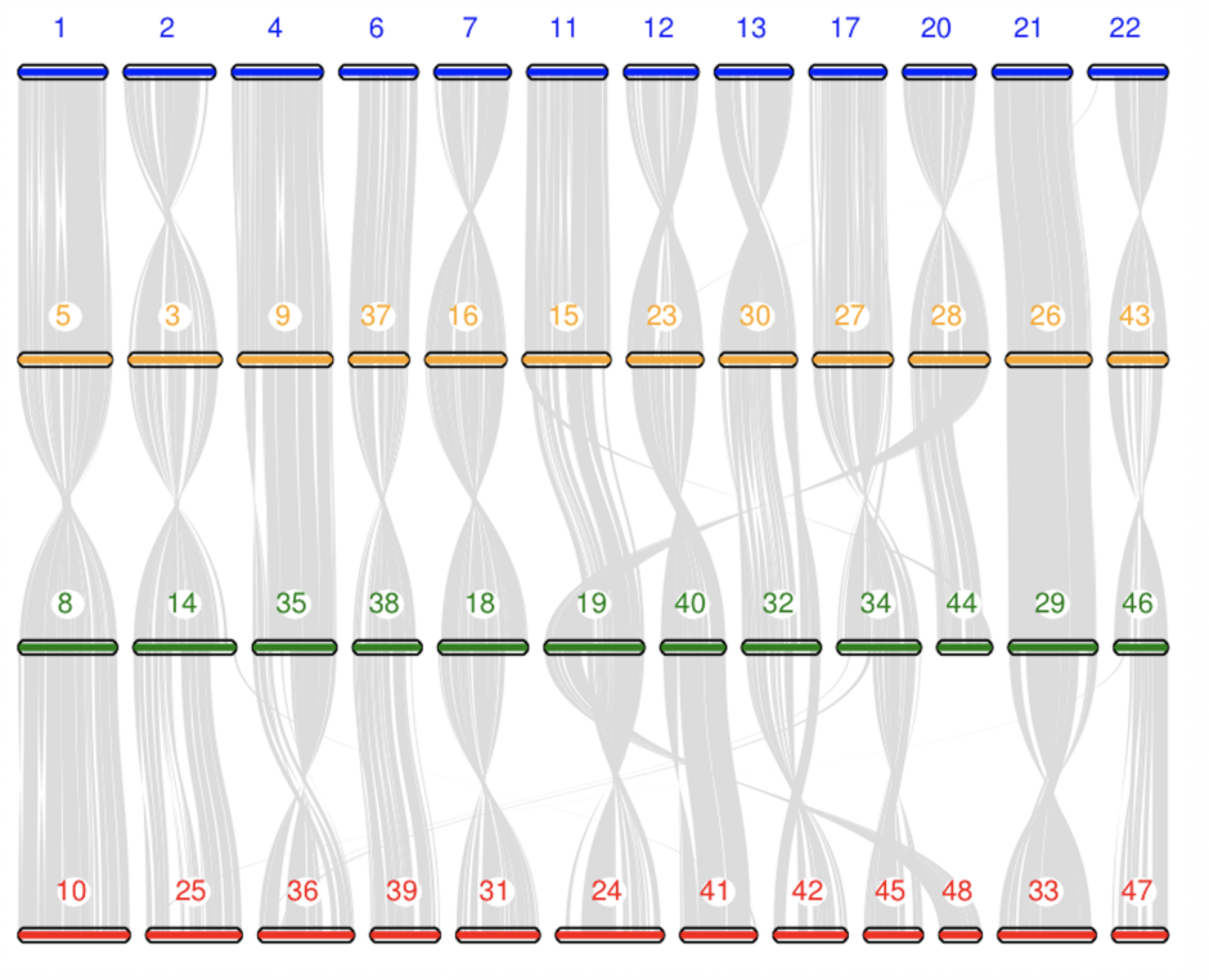


**Supplemental Figure 5:** Idiograms show which pseudomolecules (chromosome assemblies) are homoeologous by connecting gray lines. For examples, chromosomes 1, 5, 8 and 10 are homoeologous in the Draper genome assembly [(Colle et al. 2019)](https://paperpile.com/c/AK2dZ7/4O1i).


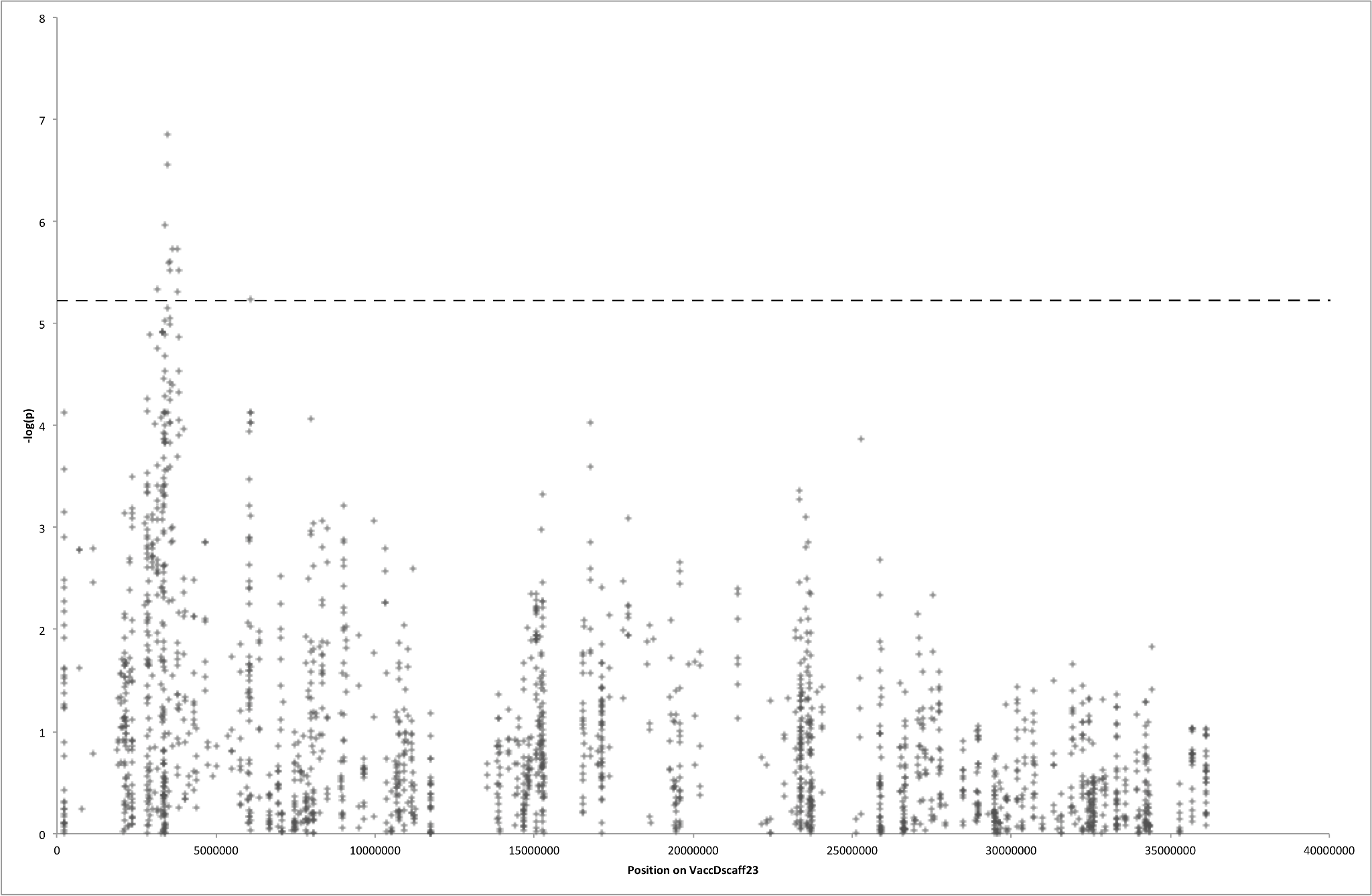


**Supplemental Figure 6:** GLM association results for chromosome 23 showing the presence of to peaks that exceed the significance threshold, one more prominent one falling before the 5,000,000 marker on the x-axis, and the other falling after the 5,000,000 marker.


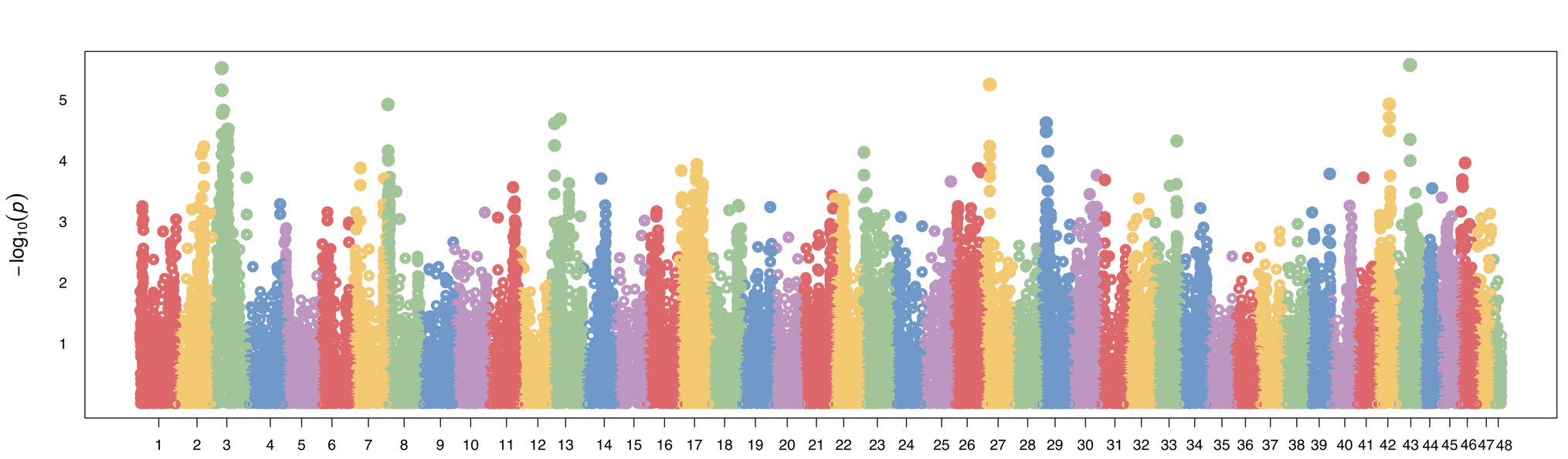


**Supplemental Figure 7:** GLM association results with a binarized phenotype 0-1:0 >1:1. No significant association peaks are detected after phenotype binarization.

| **Identifier** | **2020** | **2021** |
| --- | --- | --- |
| **136** | 0 | N/A |
| **170** | 0 | 0 |
| **302** | 0 | N/A |
| **408** | 0 | 0 |
| **428** | 0 | 0 |
| **600** | 0 | 0 |
| **200** | 78.3 | 85 |
| **208** | 94.5 | 95 |
| **274** | 95 | 100 |
| **453** | 85 | 75 |
| **458** | 80 | 90 |
| **573** | 90 | 100 |

**Supplemental Table 1:** Fruit rot susceptibility percentages of individuals used for metabolite analysis from the 2020 and 2021 seasons.

**Supplemental Methods:**

**QTL mapping**

SNP markers were grouped based on physical mapping into 10-SNP windows that were subsequently aggregted by similarity and a representative tag-SNP for each aggregated linkage group was selected as the marker closest to the group average genotype. The representative markers were read into the R qtl function as proxy f2 crosses (as a consequence of the high rate of polyvalancy expected during crossing). Their locations were offset with the jittermap function and null markers were dropped. The underlying genotype was inferred with the calc.genoprob function with a 1 cM step size and an error probability of 0.01. The resulting genotypes were scanned for QTLs using either the scanone function for a single association model or the more powerful cim function for composite interval mapping both with default parameters.
